# The Genetics of Autoimmune Myositis

**DOI:** 10.3389/fimmu.2022.886290

**Published:** 2022-05-26

**Authors:** Janine A. Lamb

**Affiliations:** Epidemiology and Public Health Group, School of Health Sciences, University of Manchester, Manchester, United Kingdom

**Keywords:** myositis, idiopathic inflammatory myopathies, genetics, genetic risk factors, gene expression, pathogenesis, human leukocyte antigen

## Abstract

The idiopathic inflammatory myopathies (IIM) are rare, heterogeneous systemic autoimmune disorders, characterized by inflammation of skeletal muscle and multi-organ involvement. Studies to identify genetic risk factors and dysregulated gene expression in IIM aim to increase our understanding of disease pathogenesis. Genome-wide association studies have confirmed the HLA region as the most strongly associated region in IIM, with different associations between clinically-defined subgroups. Associated genes are involved in both the innate and adaptive immune response, while identification of variants reported in other autoimmune disorders suggests shared biological pathways. Targeted imputation analysis has identified key associated amino acid residues within HLA molecules that may influence antigen recognition. These amino acids increase risk for specific clinical phenotypes and autoantibody subgroups, and suggest that serology-defined subgroups may be more homogeneous. Recent data support the contribution of rare genetic variation to disease susceptibility in IIM, including mitochondrial DNA variation in sporadic inclusion body myositis and somatic mutations and loss of heterozygosity in cancer-associated myositis. Gene expression studies in skeletal muscle, blood and skin from individuals with IIM has confirmed the role of interferon signalling and other dysregulated pathways, and identified cell-type specific signatures. These dysregulated genes differentiate IIM subgroups and identify potential biomarkers. Here, we review recent genetic studies in IIM, and how these inform our understanding of disease pathogenesis and provide mechanistic insights into biological pathways.

## Introduction

The idiopathic inflammatory myopathies (IIM) are a rare heterogeneous group of systemic autoimmune disorders. IIM are characterized by chronic inflammation of skeletal muscle resulting in muscle weakness, and multiple other organ systems may be involved. Individuals with IIM can be classified into different disease subgroups based on clinical and/or serological criteria. Our understanding of disease pathogenesis in IIM is limited, and some individuals respond poorly to treatment, with consequently poor health outcomes. Research to identify genetic risk and protective factors, and dysregulated gene expression, has been facilitated by rapid advances in biotechnology and computational approaches. These studies can identify genetic similarities and differences between IIM subgroups, and how these ‘myositis spectrum disorders’ relate to other connective tissue diseases and autoimmune diseases more generally. This knowledge increases understanding of disease pathogenesis, and yields mechanistic insights into biological pathways and potential drug targets. Knowledge of genetic risk factors that contribute to different myositis phenotypes may enable more precise classification and inform clinical decision-making for targeted disease management and treatment. Here, we review our current knowledge of myositis genetics gained from genome-wide and targeted studies of both common and rare genetic variants, and studies of gene expression. For the purposes of this review, we consider the following IIM clinical subgroups: dermatomyositis (DM), polymyositis (PM), anti-synthetase syndrome (ASS), sporadic inclusion body myositis (sIBM), cancer-associated myositis and immune-mediated necrotising myopathy (IMNM) (1). We highlight key findings and suggest potential avenues as future research priorities.

## Large Epidemiological Studies Estimate Familial Risk and Heritability in IIM

The first large epidemiological study of familial aggregation and heritability in IIM used nationwide healthcare register data from Sweden (2). This study included 1620 individuals diagnosed with IIM between 1997 and 2016 and their first-degree relatives, compared to matched individuals without IIM. The adjusted odds ratio (OR) for individuals with IIM to have one or more first-degree relatives affected with IIM was 4.32 (95% confidence interval (CI) 2.00-9.34) compared to individuals without IIM, and was 2.53 (95% CI 1.62-3.96) in full siblings. The heritability of IIM was 22-24% among first-degree relatives and full siblings (2). These data show that having at least one first-degree relative with IIM is strongly associated with risk of IIM, and that >20% of the phenotypic variance of IIM is due to additive genetic variance in the Swedish population (2). A similar study using Swedish medical records calculated familial risk for six rheumatic autoimmune diseases (3). The standardized incidence ratio for PM/DM was 4.03 (95% CI 1.27-8.35) when any first-degree relative was considered. The familial risk of rheumatoid arthritis and systemic lupus erythematosus was also significantly increased for individuals with PM/DM (3), suggesting extensive familial poly-autoimmunity between these diseases. Due to the rarity of IIM, these studies did not have sufficient power to investigate whether familial aggregation or heritability differs between clinical subgroups or for juvenile-onset IIM. Although the heritability estimates of 22-24% for IIM (2) are lower than those reported for other autoimmune disorders such as rheumatoid arthritis or type 1 diabetes mellitus, they are considerably higher than a previous estimate of 5.5% for DM and 8.3% for PM of phenotypic variance explained by genetic variants on the Immunochip (below) (4). These lower estimates may reflect the selected content of the Immunochip and may also suggest the involvement of rare genetic variants in IIM.

## Large Scale ‘Genome-Wide’ Association Studies in IIM

Two large-scale genetic studies of IIM were conducted by the International Myositis Genetics Consortium (MYOGEN) using the Immunochip genotyping array (Illumina, USA) (5, 6). The Immunochip was designed based on genetic variants or loci associated with 12 different autoimmune or inflammatory diseases, with extended coverage across the major histocompatibility complex (MHC), so does not have full genome-wide coverage. These Immunochip studies of 2566 cases with IIM of European ancestry identified a strong signal in the MHC region meeting genome-wide significance (p< 5x10^-8^), with association to alleles of the 8.1 ancestral haplotype; HLA-DRB1*03:01 in PM and juvenile-onset dermatomyositis (JDM), and HLA-B*08:01 in adult-onset DM (6). Conditional analysis suggested that multiple alleles on the 8.1 ancestral haplotype contribute independently to disease risk. In sIBM, this first ‘genome-wide’ association study (GWAS) also identified a strong association to HLA−DRB1*03:01, and independent associations to HLA−DRB1*01:01 and HLA−DRB1*13:01; these latter two alleles were specific to sIBM (5). Homozygotes for HLA-DRB1*03:01 and HLA-DRB1*01:01 carried a lower risk for sIBM than heterozygotes, with the HLA-DRB1*03:01-HLA-DRB1*01:01 and HLA-DRB1*03:01-HLA-DRB1*13:01 genotypes observed at higher frequency than expected. Departure from additivity has been observed for other autoimmune disorders, and may reflect increased antigen binding and presentation in heterozygous individuals (7). Further analysis showed that alanine or serine at amino acid position 57 of HLA-DQB1 confer increased risk of DM. Asparagine or arginine at positions 77 and 74 respectively of HLA-DRB1 are associated with PM, while amino acid positions 11 and 26 of HLA-DRB1 are associated with IBM (5, 6). An extended genome-wide study focussed specifically on JDM identified a novel association at amino acid position 37 within HLA-DRB1, which may distinguish juvenile- from adult-onset DM (8). These amino acids may be more strongly associated than classical HLA alleles, and affect the structure of the HLA molecule to influence antigen recognition and presentation of peptides to the immune system. However, this analysis approach (9) does not capture non-classical *HLA* genes, and other genes within the MHC, such as copy number variants of complement genes (10), may contribute to IIM disease risk.

Outside the HLA region, genes involved in both the innate and adaptive immune response were implicated from these Immunochip studies, including *STAT4* (Signal Transducer and Activator of Transcription 4)*, TRAF6* (TNF Receptor Associated Factor 6), *UBE2L3* (Ubiquitin Conjugating Enzyme E2 L3) and *PTPN22* (Protein Tyrosine Phosphatase Non-Receptor Type 22) in IIM. *PTPN22* [rs2476601 (R620W)] reached genome-wide significance specifically in the PM subgroup [OR (95%CI) 1.58 (1.38-1.81)] (6). *PTPN22* and *STAT4* affect T cell signalling, while *TRAF6* and *UBE2L3* affect B cells and nuclear factor kappa-β (NF-κB) signalling in response to pro-inflammatory cytokines. In the relatively small cohort of 252 patients with sIBM, a strong association to chromosome 3 [OR (95% CI) 0.42 (0.29-0.60)] implicated a frameshift mutation (rs333) in the *CCR5* (C-C motif chemokine receptor 5) gene as the possible functional variant (5). CCR5 binds pro-inflammatory chemokines, and a non-functional CCR5 receptor and/or decreased expression of *CCR5* may reduce migration of T cells into muscle fibres.

A recent imputation analysis based on the MYOGEN Immunochip data was conducted to identify novel associated variants, validate existing signals and to fine-map existing associations in IIM (11). The HLA locus was again strongly associated, with the strongest subgroup association observed in the 311 cases with anti-Jo1 autoantibodies [OR (95% CI) 5.15 (4.21-6.32)]. Four non-HLA loci were identified at genome-wide significance, including *NAB1* (NGFI-A binding protein 1) in the PM subgroup [OR (95% CI) 1.41 (1.24-1.60)]. The associations to *STAT4* and *DGKQ* (diacylglycerol kinase theta) in IIM, *NAB1* and the *FAM167A-BLK* (family with sequence similarity 167 member A - BLK proto-oncogene, Src family tyrosine kinase) region in PM, and *CCR5* in sIBM were more significant than previously reported, and several associations were localized to single genes, although no significant associations outside the HLA region were identified in the anti-Jo1 subgroup. The associated variants were significantly enriched in regions of open chromatin in primary CD19+ B cells and CD3+ T cells, implicating functionally relevant cell types in IIM, although it is worth noting that variants were imputed and identified from an immune-focussed array (11).

To investigate the possible shared genetic basis of four seropositive systemic rheumatic diseases, genome-wide data from individuals of European ancestry with IIM, rheumatoid arthritis, systemic lupus erythematosus or systemic sclerosis was meta-analysed (12). This systematic approach leveraged power across studies to identify 26 genome-wide significant non-HLA loci associated with at least two diseases, emphasizing the shared genetic relationship between these diseases, and supporting the epidemiological study described previously (3). Five loci with a role in immune processes, *NAB1, KPNA4-ARL14* (karyopherin subunit alpha 4 - ADP ribosylation factor like GTPase 14)*, DGQK, LIMK1* (LIM domain kinase 1) and *PRR12* (proline rich 12), had not been associated at genome-wide significance with these diseases previously. The associated variants were predicted to have functional effects in disease-relevant immune cells. Proteins regulated by associated variants were also enriched as targets for drugs at any stage of development for treatment of these diseases, indicating the potential for drug repositioning between these disorders where there are few existing specific treatments (12).

Trans-ancestry GWAS have generated significant insights in several autoimmune disorders, increasing power and breaking down ancestry-specific linkage disequilibrium to localise association signals, but have not yet been conducted in IIM. A IIM GWAS reported in an Asian population included 576 adults from Japan, including 33 individuals with clinically amyopathic dermatomyositis (CADM) (13). Notably, there were no significant associations, including to the HLA region, in IIM or the PM or DM subgroups. However, this study identified genome-wide significant association to a variant of the *WDFY4* (WDFY family member 4) gene in CADM [OR (95% CI) 3.87 (2.23-6.55)], with nominal association of neighbouring *WDFY4* variants in 21 adults with CADM of European ancestry. WDFY4 interacts with pattern recognition receptors, and the significantly associated splicing variant was shown *in vitro* to create a new coding exon and a truncated WDFY4 protein isoform, which increased NF-ĸB activity, altered MDA5 (melanoma differentiation-associated gene 5) signalling and increased MDA5-induced apoptosis (13). Anti-MDA5 autoantibodies are more often found in amyopathic patients with rapidly progressive interstitial lung disease. This strong association in the Japanese population may therefore reflect different anti-MDA5 autoantibody frequency in the Japanese compared to European CADM samples (13/18 (72%) vs 0/17 (0%) positive of those tested) (13), and different geographic viral triggers (14), given the role of MDA5 as a cytoplasmic viral dsRNA sensor in the host anti-viral response.

A pharmacogenomics study using the Estonian Biobank integrated whole genome sequencing with drug prescription and adverse drug effect data from electronic health record databases to identify genetic variants associated with drug response (15). This population-based study of >2200 individuals identified a novel intronic variant of the Catenin alpha 3 (*CTNNA3*) gene associated with myositis and myopathies in individuals taking nonsteroidal anti-inflammatory oxicams (15). The encoded protein plays a role in cell-cell adhesion in muscle cells, so these findings on drug-induced myositis may have relevance to idiopathic inflammatory myopathies.

**Figure 1:**
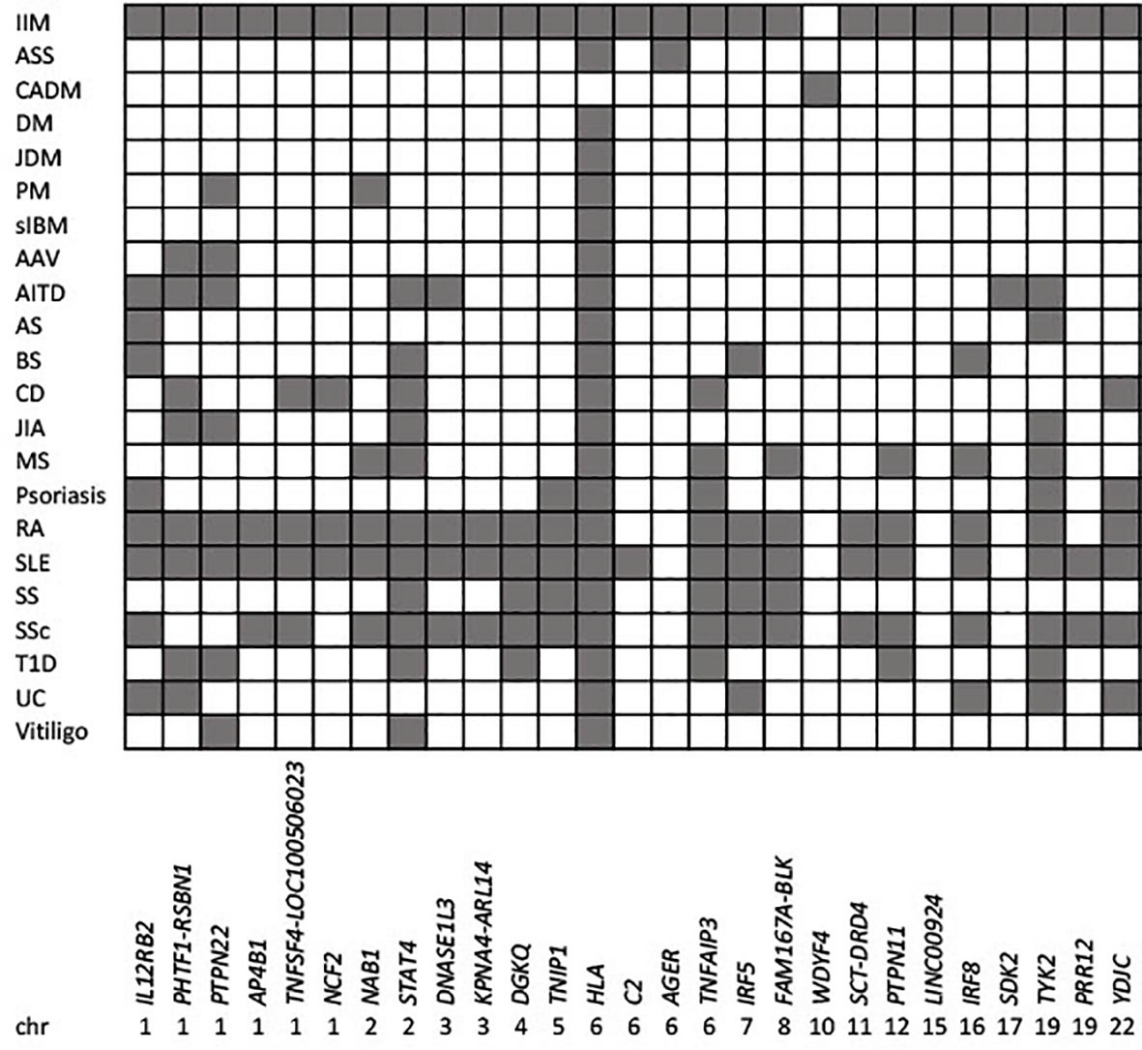
Genetic overlap between idiopathic inflammatory myopathies and other autoimmune diseases. IIM associated loci meeting genome-wide significance (p<5x10^-8^) for IIM as a whole, or for different clinical subgroups are indicated by the reported gene/s, and ordered by chromosomal location (x axis). Shaded squares show loci associated with IIM, and other autoimmune traits (y axis) reported in the GWAS catalogue v1.0.2 (www.ebi.ac.uk/gwas/home; Experimental Factor Ontology “Immune system disorder”). AAV, Anti-neutrophil cytoplasmic autoantibody (ANCA)-associated vasculitis; AITD, autoimmune thyroid disease; AS, ankylosing spondylitis; ASS, anti-synthetase syndrome; BS, Behcet’s syndrome; CADM, clinically amyopathic dermatomyositis; CD, celiac disease; DM, dermatomyositis; IIM, idiopathic inflammatory myopathies; JDM, juvenile dermatomyositis; JIA, juvenile idiopathic arthritis; MS, multiple sclerosis; PM, polymyositis; RA, rheumatoid arthritis; sIBM, sporadic inclusion body myositis; SLE, systemic lupus erythematosus; SS, Sjogren’s syndrome; SSc, systemic sclerosis; UC, ulcerative colitis.

Overall, the different associations at some loci identified from genome-wide studies suggests different pathophysiology between clinically defined IIM subgroups. Re-analysis using the more recent EULAR/ACR classification criteria for adult and juvenile IIM (16) might be informative. The more numerous associated loci identified in PM compared to DM, based on the Immunochip content, conflicts with the current notion of PM as a ‘diagnosis of exclusion’ and DM as more homogeneous. Adult-onset DM likely constitutes a heterogeneous collection of more genetically homogeneous serology-defined subgroups, while the PM-specific *PTPN22* association may reflect a pan-autoimmune locus. Strong association to the HLA region in sIBM, irrespective of anti-cytosolic 5′-nucleotidase 1A (cN1A) autoantibody positivity, indicates an autoimmune (rather than degenerative) pathogenesis, at least in some sIBM patients. Overall, the studies in populations of European ancestry described here indicate that associations to the HLA region show the largest effect size in serology-defined subgroups, followed by sIBM and PM, with adult- and juvenile-onset DM showing weaker HLA associations. Given the clinical heterogeneity of IIM, ongoing studies of clinically defined endophenotypes with significant morbidity, such as calcinosis in JDM, are likely to be informative. Furthermore, the finding that IIM-associated variants overlap with those reported in several other autoimmune disorders, including celiac disease, rheumatoid arthritis, systemic lupus erythematosus and type 1 diabetes (**Figure 1**), is consistent with an earlier GWAS in DM (17) and again suggests shared biological pathways.

## HLA Associations can Discriminate Serology-Defined Subgroups

The identification of differentially associated HLA alleles and amino acids in clinically-defined subgroups lends support to IIM as a heterogeneous collection of myositis spectrum disorders. To address this question, international collaboration through MYOGEN enabled identification of a significant number of individuals positive for mutually exclusive myositis-specific autoantibodies (18). Targeted analysis of the HLA region using SNP2HLA (9) identified study-wide significant association (p<2.9 x 10^-5^) to classical *HLA* alleles and specific amino acids, for eight of twelve serology-defined subgroups with >10 individuals (18). Associations to the 8.1 ancestral haplotype (including HLA−DRB1*03:01 and HLA−B*08:01) were identified for the anti-Jo1, anti-PM/Scl and anti-cN1A subgroups, with associations independent of this haplotype for anti-Mi-2 (HLA−DRB1*07:01) and anti-HMGCR (HLA−DRB1*11:01). Amino acid residues were often more strongly associated than classical *HLA* alleles, indicating key positions within the HLA molecule that may increase risk, such as amino acid position 74 in *HLA-DRB1* for anti-Jo-1, PM/Scl, cN1A and SRP. This amino acid faces inwards of the peptide binding groove and has been associated with risk for development of several autoimmune diseases (19). Notably, amino acids, but not classical *HLA* alleles, were significantly associated for the anti-SRP and anti-SAE autoantibody subgroups (18), possibly reflecting amino acid residues shared across multiple HLA alleles. Individuals with anti-TIF1 autoantibodies were associated with *HLA-DQB1.* However, haplotypic associations differed with age of onset, with the strongest associations to HLA-DQB1*02:01 (8.1 ancestral haplotype) and HLA-DQB1*02:02 in juvenile- and adult-onset DM respectively (18). Different associations also have been reported for a small number of individuals with anti-HMGCR-positive juvenile- (HLA−DRB1*07:01) vs adult-onset (HLA−DRB1*11:01) IMNM (20, 21).

Autoantibody-phenotype associations appear to be broadly consistent across ethnic groups in IIM. A study of 179 Korean adults with IIM investigated *HLA-DRB1* and *HLA-DPB1* associations with anti-MDA5, anti-aminoacyl-tRNA synthetase (ARS), anti-Mi2, anti-TIF1 and anti-SRP autoantibodies (22). Association of the most common autoantibody, anti-MDA5, was observed to HLA-DRB1*12:02 [OR (95% CI) 5.46 (2.67-11.20)] (22). Although no significant associations of the anti-MDA5 subgroup were observed in the MYOGEN study (18), anti-MDA5 is less frequent, and HLA-DRB1*12:02 is rare, in populations of European ancestry (23). Conversely, the 8.1 ancestral haplotype is rare in Asian populations, and anti-ARS autoantibodies were associated with HLA-DRB1*08:03 in the Korean population [OR (95% CI) 4.15 (1.89-9.09)] (22). Association of anti-Mi-2 to HLA-DRB1*07:01 [OR (95% CI) 10.23 (3.81-27.51)] (22) was consistent with the European ancestry population (22). Studies of specific *HLA* alleles and autoantibody subgroups carried out in Vietnamese and Chinese Han populations have reported different, and sometimes conflicting, associations (24–26).

Overall, the strong associations observed for some autoantibody subgroups despite a relatively small cohort size suggests homogeneity and argues for a serology-defined nomenclature in IIM. The lack of significant associations observed for the anti-NXP2 and anti-MDA5 autoantibody positive subgroups in Caucasians (18) may reflect smaller sample size, or a more heterogeneous cohort containing juvenile- and adult-onset individuals with these autoantibodies. Well-powered trans-ancestry studies are needed to refine *HLA* allele and amino acid associations and determine whether amino acid associations are shared across ethnic groups. Further studies should investigate the possible interaction of the HLA region with environmental risk factors, and determine whether amino acid associations are shared across diseases for autoantibodies such as PM/Scl.

## Mitochondrial DNA Variation Is Identified in Sporadic Inclusion Body Myositis

Mitochondrial pathology is frequently observed in sIBM muscle, presenting as ragged red fibres and cytochrome c oxidase (COX)-deficient muscle fibres causing mitochondrial respiratory chain deficiency. Deep sequencing of mitochondrial DNA (mtDNA) identified an increased frequency of somatic large mtDNA deletions and duplications correlating with a higher heteroplasmy level in sIBM muscle compared to age-matched controls (27). Recurrent deletions or duplications in sIBM patients were mainly localised to three mtDNA regions. There was also an increase in the number of somatic coding single nucleotide variants, and a reduced mtDNA copy number, in sIBM muscle (27). Previous studies have reported that large scale mtDNA deletions in COX-deficient muscle fibres in sIBM correlate with T-lymphocyte infiltration and muscle fibre atrophy (28, 29). These findings suggest an accelerated mitochondrial muscle aging process in sIBM, possibly related to chronic inflammation.

## Rare Genetic Variation Likely Plays a Role in IIM Disease Risk

The fact that IIM are rare heterogeneous disorders suggests the potential role of rare genetic risk factors, at least in a subset of individuals. Rare genetic variants (minor allele frequency <1%) are generally not well captured by genome-wide genotyping arrays, and may require alternative approaches for detection, such as next generation sequencing. Targeted DNA sequencing of ~1900 immune-related genes was used to investigate the contribution of rare and common genetic variation in a Scandinavian case-control cohort including 454 IIM cases (30). Gene-based aggregate testing, rare variant and enrichment analyses were applied. *IFI35* (interferon-induced protein 35) was identified as a significant novel genetic risk locus for IIM, suggesting type I interferon activation and a possible regulatory effect of associated variants on the skeletal muscle-specific gene *PTGES3L* (prostaglandin E synthase 3 like). Aggregate genetic associations to *AGER* (advanced glycosylation end-product specific receptor) and proteasomal genes *PSMB8* and *PSMB9* (proteasome 20S subunit beta 8/9) were identified in individuals with anti-synthetase syndrome (ASS). An increased burden of rare non-coding variants, and synonymous variants particularly in genes in the JAK-STAT signalling pathway, was also identified in individuals with IIM irrespective of myositis subtype, although there was no enrichment of missense coding variants (30).

The existence of hereditary diseases that mimic sIBM supports the contribution of rare genetic variation to this IIM subtype. Guttches *et al. *(31) identified a possible role of rare missense variants in the *FYCO1* (FYVE and coiled-coil domain autophagy adaptor 1) gene in sIBM using whole exome sequencing. Similarly, sequencing of candidate genes involved in neuromuscular or neurodegenerative diseases related to sIBM, and whole exome sequencing of genes encoding proteins overrepresented in rimmed vacuoles from skeletal muscle, identified rare variants in the *VCP* (valosin containing protein)*, SQSTM1* (sequestosome1) and *FYCO1* genes in sIBM patients (32, 33). These findings suggest impaired protein homeostasis, autophagy and proteasomal degradation in sIBM, again supporting both degenerative and inflammatory pathways in sIBM pathogenesis.

Individuals with IIM may present with temporally associated cancer, particularly in adult-onset DM with anti-TIF1 (transcription intermediary factor 1) autoantibodies. A mutation in the tumour may cause an increase in neo-antigen expression, which triggers an anti-tumour response and paraneoplastic autoimmune rheumatic disease (34). Supporting this hypothesis, an increased rate of somatic mutations and loss of heterozygosity was observed in the genes encoding TIF1 in tumour DNA from anti-TIF1 positive cancer-associated myositis patients compared to anti-TIF1-negative myositis patients (35). These mutations were not observed in blood genomic DNA. Similar findings have been reported in the polymerase (RNA) III subunit A (*POLR3A*) locus encoding RPC1 in tumour DNA from autoantibody positive patients with systemic sclerosis, but not in patients without autoantibodies to RPC1, or in peripheral blood (36).

Collectively, these findings support the contribution of both rare and common genetic variation to disease susceptibility in IIM. However, it is not yet clear where some individual patients with IIM, or different IIM subgroups, sit on the Mendelian - polygenic, complex disease axis.

## Sex Chromosome Aneuploidies May Contribute to Disease Risk in IIM

Many autoimmune diseases show a gender bias with females more typically affected, as seen in PM and DM. The reason for this gender bias is not known, but hypotheses include a gene dosage effect of genes escaping X-inactivation [such as Toll-like receptor 7 (*TLR7*) and TLR adaptor interacting with endolysosomal SLC15A4 (*TASL* or *CXorf21*)] in 46,XX females, compared to 46,XY males. In keeping with this hypothesis, an increased rate of X chromosome aneuploidies, particularly 47,XXY (Klinefelter’s syndrome) and 47,XXX, was reported in individuals with systemic lupus erythematosus or Sjögren’s syndrome (37, 38). To investigate the frequency of X chromosome aneuploidies in IIM, genotyping data from the MYOGEN Immunochip studies (5, 6) was analysed. Elevated rates of 47,XXY were observed in males with PM or DM compared to controls and to published live birth rates; a finding that was replicated in the Japanese GWAS data (39). Five of the seven PM or DM 47,XXY cases had cancer-associated myositis (39). Notably, the prevalence of 47,XXY was particularly elevated in sIBM, together with an increased frequency of 47,XXX in females with sIBM (39), despite sIBM showing the opposite gender bias to PM and DM, with more males affected. Although a causal relationship has not been established, these findings of increased 47,XXY and 47,XXX in IIM may offer mechanistic insights in a subset of patients, and in IIM more generally.

## Gene Expression Studies Differentiate IIM Subgroups and Identify Potential Biomarkers

Complementing studies of genetic variation, an increasing number of studies of gene expression in IIM have been conducted in recent years. By applying array, RNA sequencing, qRT-PCR and pathway and network analysis approaches, altered gene expression has been investigated in clinically and serology-defined IIM subgroups in skeletal muscle, skin, blood and specific cell populations.

Several systemic autoimmune connective tissue diseases including IIM show a strong type I interferon (IFN) signature in blood and target organs which has been associated with disease activity. Signatures of type I and type II IFN pathway activation were therefore investigated using RNA sequencing of muscle biopsy samples from different subtypes of myositis, compared to normal muscle biopsies (40). Expression of type I IFN-inducible genes was high in DM, moderate in ASS and low in sIBM and IMNM, whereas type II IFN-inducible gene expression was high in DM, sIBM and ASS, but low in IMNM, suggesting differential interferon pathway activation across myositis subtypes. The type I IFN signature was also up-regulated in DM-specific autoantibody subgroups. Expression of IFN-inducible genes correlated with genes involved in inflammation and muscle regeneration, but not genes encoding mature muscle structural proteins (40). Similarly, although positivity for a myositis-specific autoantibody did not correlate with expression of the corresponding autoantigen in myositis muscle biopsies, myositis autoantigens were highly expressed during muscle regeneration. Myositis autoantigen expression correlated with regeneration genes, and inversely correlated with markers of mature muscle (41).

IFN and IFN-regulated gene expression has also been studied in juvenile-onset myositis. Expression of IFN-induced 15-kd protein (*ISG15*), a negative regulator of type I IFN, in deltoid muscle biopsy samples discriminated juvenile IIM patients from those with non-immune myopathies, and was highest in anti-MDA-5 positive JDM patients (42). Systemic IFNα protein concentrations were also higher in anti-MDA5 positive juvenile patients (43). Type I interferonopathies are genetically heterogeneous Mendelian disorders with persistent upregulation of type I IFN (44). A high prevalence of overlapping autoimmune disease is reported in some interferonpathies, and disorders such as chronic atypical neutrophilic dermatosis with lipodystrophy and elevated temperature (CANDLE) syndrome and STING-Associated Vasculopathy with onset in Infancy (SAVI) share clinical and immunological features with JDM. The IFN-regulated gene signature from peripheral blood was found to be lower in JDM than CANDLE and SAVI patients, but overlapped with SAVI particularly in anti-MDA5 positive JDM, and correlated moderately with disease activity measures (45).

Targeted studies of the mRNA and protein levels of immune-related molecules have also been carried out in both juvenile- and adult-onset IIM, and in serology-defined subgroups. Serum levels of galectin-1, galectin-9, CXCL10 (C-X-C motif chemokine 10 precursor) and TNFRII (TNF receptor superfamily member 1B) proteins correlated with severity of disease activity in treatment-naïve JDM patients (46). Galectin-9 mRNA was also increased in peripheral blood mononuclear cells from DM and anti-MDA5 positive adult-onset patients and correlated with the type I IFN inducible genes *MX1* (MX dynamin like GTPase 1) and *IFIH1* (interferon induced with helicase C domain 1) (47). Similarly, serum levels of galectin-9 protein were increased and correlated with disease activity (47).

In sIBM, studies of muscle using gene expression microarrays identified a muscle cytotoxic T-cell signature and a highly differentiated CD8^+^ T-cell signature compared to normal samples, and distinct from other forms of IIM and other autoimmune and non-immune muscle diseases, including muscular dystrophies and mitochondrial myopathies (48). Expression of markers of T-cell autoimmunity were also higher in sIBM, including T-cell related cytokines, interferon-gamma induced chemokines and upregulation of the immunoproteasome (48).

Further analysis of muscle biopsy RNAseq data identified upregulated gene expression networks in IIM including muscle regeneration, acute phase response and neutrophil degranulation, while some pathways such as myofilaments and mitochondrial envelope were suppressed (49). Myositis subtype-specific networks were also identified, including type I IFN signalling and Titin in DM, RNA binding in ASS and vasculogenesis in sIBM, suggesting different pathophysiological mechanisms (49). Application of machine learning and deep learning approaches showed that patients could be assigned to clinical groups and further molecular sub-clusters based on their muscle biopsy transcriptome profile (49, 50). Similarly, unsupervised clustering of gene expression data generated using the NanoString^®^ nCounter PanCancer Immune Profiling Panel™ stratified anti-TIF1 and anti-Mi-2 positive DM patients, and identified a type I IFN signature in anti-Mi-2 muscle biopsy samples (51).

Finally, the transcriptional profile of DM lesional skin biopsies was compared to cutaneous lupus erythematosus, as these are often difficult to distinguish histologically (52). Dysregulated type I IFN signalling was common to both diseases, but DM skin lesions could be differentiated from cutaneous lupus erythematosus by a biomarker panel of five genes including *IL18* (interleukin 18). Based on single cell RNA sequencing, keratinocytes were identified as the possible source of *IL18* upregulation in DM skin (52).

Overall, these gene expression studies are adding to our knowledge of IIM pathogenesis, linking genetic variation to mRNA expression and protein translation, and providing mechanistic insights, potential therapeutic targets and biomarkers for monitoring disease activity.

## Molecular Characterisation of IIM

More precise molecular classification of patients with IIM into well-characterised subgroups should inform clinical decision-making for more targeted management and treatment of disease. This may increase the success of clinical trials. Classification ‘labels’ are important in this respect, and parallels can be drawn to oncology where common cancers such as melanoma and non-small cell lung cancer are now collections of rare cancers. Our interpretation of some current findings may be altered by application of more recent classification criteria to define IIM clinical subgroups (16). Alternatively, a serology-defined nomenclature could be used. However, this is not without challenges; approximately one-third of patients with IIM are known myositis-specific or -associated autoantibody negative, many individuals have as yet unidentified autoantigens, and there is no consistent and accepted approach for autoantibody identification.

Increasingly, researchers are conducting more integrated multi-omics profiling, including genetic, genomic, proteomic, metabolomic, epigenetic and microbiome data. A molecular taxonomy to classify patients with seven systemic autoimmune diseases was recently reported (53). This study used unsupervised clustering of whole blood transcriptome andmethylome data, and clinical, serological (including autoantibodies and cytokines) and cellular features from flow cytometry (53). Four clusters of patients were defined independent of clinical diagnosis, with three of the clusters representing inflammatory, lymphoid and interferon molecular signatures, suggesting different disease mechanisms. Furthermore, in a separate cohort, the large majority of patients remained in the same cluster after longitudinal follow-up. Notably, *HLA* genetic associations in this study related to molecular clusters, rather than clinical diagnosis (53). Similar approaches using high-dimensional data and integrated datasets could be informative, but have not yet been applied at scale, in IIM.

## Looking Forwards in IIM Genetics Research

Collectively, the genetic studies reviewed here have increased our knowledge of the pathogenesis of these rare, heterogeneous immune-mediated inflammatory disorders. However, it is not yet clear where some individual patients with IIM, or different IIM subgroups, sit on the Mendelian - polygenic, complex disease axis. We might expect a juvenile-onset disorder such as JDM to have a higher burden of rare, more penetrant, risk alleles. Advances in genetic technology will enable investigation of larger numbers of patients through whole exome and whole genome sequencing studies to better characterise the role of rare and non-coding genetic variants in IIM. Genetic studies can also provide insight into the autoimmune - auto-inflammatory axis and inflammatory versus degenerative pathology, including the role of mitochondria, autophagy and the immunoproteasome, in different IIM subgroups.

Single-cell transcriptomic approaches will allow better characterisation of gene expression in different cell types, unmasking cellular heterogeneity and addressing the multi-organ pathology and inflammatory and degenerative components of IIM. Towards this aim, numerous analytical approaches now exist based on known cell-type gene expression signatures to estimate the abundance of constituent cell types in a mixed cell population from bulk RNA-sequencing data, to distinguish between changes in cell-type composition and changes in cell state.

Novel approaches applied to existing IIM data might be instructive. For example, a shrinkage approach using GWAS summary statistics increased power to identify novel associations, look for similarities and differences, and biologically characterise multiple immune-mediated diseases (54). Other statistical approaches such as machine learning, Mendelian randomization and polygenic risk score analysis are currently being applied in IIM. Studies can also leverage public and private biobanks and repositories including genetic data such as UK Biobank, gnomAD (55), FinnGen and 23andMe.

The role of environmental factors, and gene-environment interactions, also needs further investigation, given the involvement of smoking (56, 57) and statins, and the possible role of viruses and seasonal variation in IIM. Overall, the validity and clinical utility of genetic findings will rely on our ability to translate these biomarkers into improved patient classification, disease management and treatment, and improved health outcomes for individuals with IIM.

## Author Contributions

The author confirms being the sole contributor of this work and has approved it for publication.

## Conflict of Interest

The author declares that the research was conducted in the absence of any commercial or financial relationships that could be construed as a potential conflict of interest.

## Publisher’s Note

All claims expressed in this article are solely those of the authors and do not necessarily represent those of their affiliated organizations, or those of the publisher, the editors and the reviewers. Any product that may be evaluated in this article, or claim that may be made by its manufacturer, is not guaranteed or endorsed by the publisher.
